# Supported Internet-Delivered Cognitive Behavioral Therapy Programs for Depression, Anxiety, and Stress in University Students: Open, Non-Randomised Trial of Acceptability, Effectiveness, and Satisfaction

**DOI:** 10.2196/11467

**Published:** 2018-12-14

**Authors:** Jorge E Palacios, Derek Richards, Riley Palmer, Carissa Coudray, Stefan G Hofmann, Patrick A Palmieri, Patricia Frazier

**Affiliations:** 1 E-mental Health Research Group School of Psychology University of Dublin, Trinity College Dublin Dublin Ireland; 2 Clinical Research & Innovation SilverCloud Health Dublin Ireland; 3 Department of Psychology University of Minnesota Minneapolis, MN United States; 4 Department of Psychological and Brain Sciences Boston University Boston, MA United States; 5 Summa Health Traumatic Stress Center Summa Health System Akron, OH United States

**Keywords:** depression, anxiety, cognitive therapy, students

## Abstract

**Background:**

Many university campuses have limited mental health services that cannot cope with the high demand. One alternative is to use internet-delivered cognitive behavioral therapy (iCBT) as a way of tackling barriers such as lack of availability and scheduling issues.

**Objective:**

This study aimed to assess feasibility, acceptability, effectiveness, and satisfaction of a supported iCBT intervention offering 3 programs on depression, anxiety, and stress to university students. The design was an open or nonrandomized feasibility trial.

**Methods:**

Participants were recruited from 3 counseling centers at a large midwestern University in the United States. Those agreeing to take part chose 1 of 3 iCBT programs—*Space from Depression*, *Space from Anxiety*, or *Space from Stress* —all comprised 8 modules of media-rich interactive content. Participants were supported throughout the trial by a trained professional. The Patient Health Questionnaire 9 (PHQ-9), Generalized Anxiety Disorder 7 (GAD-7) questionnaire, and stress subscale of the Depression Anxiety and Stress Scale (DASS-21) were completed at baseline, 8 weeks, and 3-month follow-up. A Satisfaction With Treatment (SAT) questionnaire was completed at 8 weeks, and qualitative interviews were completed by a subsample of participants at 3 months.

**Results:**

A total of 102 participants were recruited, with 52 choosing *Space from Anxiety*, 31 choosing *Space from Depression*, and 19 choosing *Space from Stress*. Mixed-effects models showed a significant decrease in symptoms of depression (*F*_4_=6.36, *P*<.001), anxiety (*F*_4_=7.97, *P*<.001), and stress (*F*_4_=8.50, *P*<.001) over time across all 3 programs. The largest decreases in PHQ-9 scores at 8 weeks were among participants who chose the *Space from Depression* program (*d*=0.84); at 3 months, the largest decreases in PHQ-9 scores were among those who chose the *Space from Stress* program (*d*=0.74). The largest decreases in GAD-7 scores were among those who chose the *Space from Anxiety* program (*d*=0.74 at 8 weeks and *d*=0.94 at 3 months). The largest decrease in DASS-21 stress subscale scores was among those who chose the *Space from Stress* program (*d*=0.49 at 8 weeks and *d*=1.16 at 3 months). The mean time spent using the platform per session was 27.4 min (SD 33.8), and participants completed 53% (SD 37.6) of the total program content on average. Most (37/53, 69%) participants found the programs helpful or very helpful and liked the convenience and flexibility of the intervention. Qualitative interviews (n=14) indicated the intervention met students’ expectations, and they saw it as a valuable complement to face-to-face treatment.

**Conclusions:**

The iCBT programs tested in our study appear to be feasible, acceptable, and effective in a university environment. Participants described the benefits of having a flexible, supported Web-based intervention available on campus. Larger trials should be conducted to further test the effectiveness of supported Web-based interventions that give students a choice of program depending on their symptom profile.

## Introduction

### Background

Within the university student population, depression and anxiety are among the most prevalent mental health conditions [1]. The 2017 American College Health Survey found that 61% of college and university students reported experiencing *overwhelming* anxiety on at least one occasion in the previous 12 months, and 39% reported feeling so depressed that it was *difficult to function* at least once in the same time period [2]. These numbers reflect the difficulties young people might experience on entering higher education, including academic pressure [3,4], managing social demands [5], and developmental changes that might give rise to mental health difficulties [1]. Counseling center directors report that there has been an increase in recent years in the number of students seeking help for serious psychological problems [6].

Current mental health resources on campus often do not adequately address the needs of the student population [6]. For example, because of the increased demand for services, counseling centers often have long waiting lists [7]. In addition, many students with mental health issues do not seek services [8]; the primary barriers include stigma, lack of time, and scheduling concerns [9].

Internet-delivered mental health interventions might be one way to address the increased demand for mental health care. A recent meta-analysis of 48 studies supported the effectiveness of technology-based mental health interventions (mostly delivered via computer) in college student samples [10] (relative primarily to wait-list control groups). Most Web-based mental health interventions are based on cognitive behavioral therapy (CBT), which is an empirically supported treatment for many mental health disorders [11]. Although interventions can be self-guided, those that are clinician-supported are generally more effective than those that are self-guided [10,12]. Web-based mental health interventions can be disseminated widely and, therefore, might address the limited availability of in-person counseling services. In addition, because these interventions can be accessed remotely and at the students’ convenience, they allow for flexibility in working around busy schedules and alleviate concerns about stigma.

Although internet-delivered CBT (iCBT) has been shown to be effective for college students [10], some gaps remain in our understanding of how best to implement these interventions on campus. Many studies have used convenience samples of undergraduate students, particularly students in psychology classes [10]; however, few studies have tested the feasibility and effectiveness of iCBT when implemented as part of a campus service delivery system. In addition, previous meta-analyses have highlighted the need for more user feedback from interviews and other qualitative methods to better tailor Web-based interventions to the needs of college students [13]. For example, 1 qualitative study found that college student users of a Web-based stress management intervention not only liked the flexibility and anonymity of using the program but also would have liked greater individualization to the specific stressors faced by college students [14].

### Objectives

The aims of this mixed-methods study were to assess feasibility, acceptability, effectiveness, and satisfaction with iCBT interventions embedded within the care delivery system of a large university. Feasibility was defined in terms of the number of students recruited and the percentage of those that started a program. Acceptability was measured using data on usage and engagement with the intervention acquired via the Web-based system. Effectiveness was assessed using standard measures of symptoms of depression, anxiety, and stress completed at baseline, endpoint (8 weeks), and follow-up (3 months). Finally, satisfaction was measured via a questionnaire at the end of the 8-week intervention period and a semistructured phone interview at 3-month follow-up.

## Methods

### Procedures

The trial protocol containing the complete methodology has been published [15] and was approved by the University of Minnesota institutional review board (code number: 1503S64741). This was an open trial in which recruited participants were asked to choose between 3 interventions targeting depression, anxiety, or stress. Recruitment took place from October 2015 to September 2017 at the University of Minnesota-Twin Cities, which is a large urban campus, with approximately 52,000 students (6588/52,000, 12.67% international students). Approximately 15.37% (8512/52,000) of the students use campus mental health services, and there often is a wait-list.

Students were recruited through 3 campus centers: the student counseling center, a mental health clinic associated with the student health service, and the international student office. The counseling center and mental health clinic both provide individual and group psychotherapy to students, with the latter tending to serve students with more serious issues. The international student office does not provide psychotherapy but has licensed providers on staff who refer students to the other offices. Information about the programs was made available to students through posters, websites, and staff at each office. Interested students were emailed a link with more information about the program and an invitation to participate in the study. If there was a wait-list at the student counseling center or mental health clinic (which varied over time), students were told that they could access SilverCloud immediately. They were also asked to complete the Web-based program before starting face-to-face counseling. In addition, they completed the following screening measures: the Patient Health Questionnaire 9 (PHQ-9), the Generalized Anxiety Disorder 7 (GAD-7) questionnaire, and the stress subscale of the Depression, Anxiety, and Stress Scale (DASS-21). Students were provided with scores on these measures along with explanatory text (eg, “your score on the PHQ-9 would indicate mild depressive symptoms”) so that they could make an informed decision as to which of the 3 programs they wanted to select. Information regarding the programs was limited to general information regarding their basis in CBT techniques and that each was tailored to address either depression, anxiety, or stress more specifically. On choosing their preferred program, they were assigned a supporter. The intervention was intended to last for 8 weeks, with measures taken at initial assessment (screening), after 8 weeks, and at a 3-month follow-up. At 8 weeks, a Satisfaction With Treatment (SAT) questionnaire [16] was also sent as part of the follow-up. Finally, during the second year of the trial, participants were invited to complete a follow-up interview at 3 months on their perceptions, attitudes, and experiences of the intervention.

### Inclusion and Exclusion Criteria

All students over 18 years of age with computer and internet literacy enrolled at the University of Minnesota were eligible for recruitment. On completion of screening measures, students had to meet the threshold for mild depression (score of 5+ on the PHQ-9), anxiety (5+ on the GAD-7), or stress (15+ on the DASS-21 stress subscale, after multiplying score by 2). The protocol stated that participants would not be included in analyses if they did not meet the threshold for the program they chose. This occurred in only 5 cases, and in every case, the student met criteria on another measure. Therefore, these participants were not excluded from the analyses. All students were above the threshold on at least one measure.

Clients attending in-person therapy, either individually or in a group setting, were excluded from the study. Students at particular risk, based on self-harm questions, were referred to a clinician for further assessment. Throughout the trial, there was a *help* button on the program that could be accessed at any time that directed students to available resources.

### Intervention

The 3 iCBT programs (*Space from Depression*, *Space from Anxiety*, and *Space from Stress*) were delivered via a Web 2.0 platform and comprised 8 modules of media-rich interactive content. These programs were developed by SilverCloud Health, a multidisciplinary clinical, design, and development team. The SilverCloud platform employs several strategies aimed at improving the user experience, including personal, interactive, supportive, and social tools. All 3 programs incorporate core concepts of CBT, such as behavioral activation, cognitive restructuring, and challenging core beliefs. *Space from Depression* embeds these concepts into a program detailing the concept of depression, understanding negative thinking and its consequences, and including personal stories from people with a history of depression. *Space from Anxiety* includes a module on facing one’s fears and working through the hierarchy of fears. *Space from Stress* primarily focuses on improving positive well-being (eg, finding meaning, happiness, and adapting to one’s environment), addressing stress in university settings, and developing a more balanced and meaningful life. All content of these programs follows evidence-based CBT principles. Each program also incorporates introductory quizzes, videos, informational content (including stories from other users), interactive activities, and a personal journal. More detailed information on the content of the platform can be found in the published protocol [15] and in previous research [17]. The standard recommendation was to complete 1 module per week over the 8-week period, with weekly reviews provided by the supporter. Students could access the program at any moment from any computer with an internet connection.

### Support

Each study participant was assigned a staff member (who was a licensed psychologist or social worker) from the office through which they were recruited to act as supporters throughout the trial. These supporters received training in the program and how to give feedback through the Web-based system. The supporters initially sent a welcome message to the participants encouraging their use of the program and highlighting key aspects such as potential benefits and navigation through the different modules. Thereafter, the supporters logged in each week, reviewed the participants’ progress, and left feedback that included a response to the work the participants had completed that week. Participants were also able to share more information via journal entries, although this was not obligatory. The feedback given by the supporter typically took between 10 and 15 min per participant per session. Finally, if the supporter detected a long period of inactivity (more than 1 week), they sent a message prompting the student to use the program.

### Measures

All measures were completed on the SilverCloud platform. Sociodemographic data included age, gender, ethnicity, employment, relationship status, school year, and international student status. Measures gathered through active use of the platform were collected using the SilverCloud back-end data capture. These include log-ins, time spent on the platform (total and per session), modules completed, page views, and journal entries. A total of 3 primary outcome measures were used to assess effectiveness. The first was the PHQ-9 [18,19], a widely used screening tool reflecting the diagnostic criteria for depression in the Diagnostic Manual of Mental Disorders, Fifth Edition (DSM-5) [20]. The PHQ-9 comprises 9 items scored on a scale of 0 to 3, with total scores of 0 to 27. The second was the GAD-7 questionnaire [21], designed to assess anxiety per the criteria for generalized anxiety disorder in the DSM-5. It comprises 7 items scored from 0 to 3 each, with total scores of 0 to 21. The third was the DASS-21 [22] stress subscale. Items are rated on a 0 to 3 scale, with total scores ranging from 0 to 21. On all 3 measures, higher scores indicate higher symptom severity. The SAT questionnaire included 4 quantitative questions regarding satisfaction with Web-based treatment (eg, “How did this online treatment compare to previous treatments?”) measured on a 5-point scale (0=*Much better* to 4=*Not at all good*) as well as 2 open-ended questions asking participants to describe what they most and least liked about the program.

### Structured Interviews

In the second year of the trial, all students enrolled in the study were emailed to see if they were interested in completing a structured interview regarding their perceptions of the program. A follow-up email was sent if no response was received after 1 week. A total of 30 participants were contacted, and 14 agreed to be interviewed. Interviews were conducted via phone by a PhD student in counseling psychology. Interviews were recorded and then transcribed. Transcriptions were independently double-checked for accuracy. Interviews typically lasted for 10 to 15 min. In 1 case, the student declined to be interviewed and instead submitted written responses to the questions.

The interview was intended to further explore the participants’ perceptions, attitudes, and experiences regarding the intervention beyond the 2 open-ended questions on the SAT. Questions were derived by consensus among a group that included intervention supporters, counseling center administrators, a faculty member, and a graduate student. Questions focused on how and why students accessed SilverCloud (eg, “How did you hear about SilverCloud?” and “Why did you decide to use SilverCloud instead of in-person counseling?”), what they thought about specific aspects of it (eg, “Did you like that you could complete the program in any order, or would you have preferred that the program had more structure?” and “What did you think about having a supporter?”), and a more general evaluation of how well it met their needs (eg, “In general, how did you feel about using the Silver Cloud program?” and “If you have done in-person counseling before, how did it compare to SilverCloud?”).

### Data Analysis

Feasibility was measured by calculating the percentage of participants logging in to their chosen platform from those initially recruited and invited. Acceptability was assessed in terms of the total time spent on the programs, average number of log-ins, average time per session, modules completed, average page views, and average number of journal entries completed. Effectiveness was assessed using a linear mixed model (LMM) to determine the significance of changes in depression, anxiety, and stress scores from baseline to 8 weeks to 3 months in the sample as a whole. This model was then expanded to include program type to measure changes in symptoms for each of the 3 programs. LMM was used rather than the original analysis described in the protocol (repeated measures analysis of variance; ANOVA) because it is better able to handle missing data. Repeated measures ANOVA has been criticized because it uses listwise deletion [23], whereas LMM allows for more data points and subjects to be included in the model [24]. The LMM model used intent-to-treat analysis (ie, all participants who completed baseline measures were included in analyses). Missing data were estimated using maximum likelihood estimation. Within-group effect sizes (Cohen *d*) were calculated to quantify change from baseline to 8 weeks and 3 months as per the procedure suggested by Morris and Deshon [25], which takes the correlation between pre- and posttest scores into account. The reliable change index (RCI) was also calculated according to Jacobson and Truax criteria [26] for each of the outcome measures. Users achieved reliable change if their scores decreased by more than the RCI for that measure. Cohen *d* s and RCIs used data from participants who completed the 8-week and 3-month measures (ie, completer vs intent-to-treat analyses). All quantitative analyses were conducted using Stata Statistical Software 15 (StataCorp LLC, College Station, TX).

For the SAT, a descriptive analysis of the quantitative questions was followed by a thematic analysis of the qualitative questions to establish common themes regarding what participants liked most and least liked about the iCBT interventions [27]. Following an initial review of the raw data, initial themes were generated, coded, and these were then reviewed by a coresearcher.

The semistructured phone interviews were analyzed using content analysis, which is a means of identifying and interpreting patterns within a qualitative dataset. These patterns can be coded across participants and quantified [28]. The researchers chose this method to convey a sense of how frequently important themes came up in the subsample. All the responses were reviewed by 2 researchers. Each researcher first independently reviewed all the interview transcripts, noting patterns as they went. The researchers then developed codes, again independently, that described these patterns. Following this, they met in person to agree on codes and resolve discrepancies and finally sorted the responses accordingly. For example, each researcher noticed patterns in students’ responses to the question of why they chose to use SilverCloud over in-person counseling. Many mentioned that their busy schedules prevented them from attending in-person counseling and that SilverCloud was more accessible; others described being uncomfortable with the idea of seeing a counselor because the experience would be more intense. On the basis of these responses, the researchers developed 2 codes: *scheduling and convenience* and *level of comfort with in-person counseling*. The percentage of participants whose responses fell into each code was calculated.

## Results

### Feasibility

Participants were recruited between September 2015 and May 2017. Those who expressed interest (n=182) were sent a link with more information and invited to use the Web-based platform. Of those invited, 56.0% (102/182) opted into the study (75 from student counseling services, 20 from the mental health clinic, and 7 from the international student office) and engaged in their chosen program. The majority chose *Space from Anxiety* (52/102, 51.0%), a smaller percentage chose *Space from Depression* (31/102, 30.4%), and a minority chose *Space from Stress* (19/102, 18.6%). The majority screened positive for all 3 conditions (57.8%, 59/102). Of these, 32 (32/59, 54%) chose *Space from Anxiety*, 14 (14/59, 24%) chose *Space from Depression*, and 13 (13/59, 22%) chose *Space from Stress*. Of those who screened positive for 2 conditions (24/102, 23.6%), 15 (15/24, 62%) chose the *Space from Anxiety* program, 7 (7/24, 29%) chose *Space from Depression*, and 2 (2/24, 8%) chose *Space from Stress*. In total, 95.1% (97/102) chose programs for which they screened positive. Our goal was to recruit 35 in each arm. We fell short of that goal, especially for *Space from Stress*, but opted not to recruit data for an additional academic year to reach the target recruitment goal for that program. Among the 102 participants, 52.0% (53/102) completed outcome assessments at 8 weeks. Of those 53, 79% (42/53) completed assessments at 3 months. Furthermore, 8 participants provided data at 3 months but not at 8 weeks. The full flowchart from recruitment to allocation and follow-up is shown in the Consolidated Standards of Reporting Trials diagram (Multimedia Appendix 1). Little Missing Completely at Random (MCAR) test for missing data was nonsignificant for the PHQ-9 (*P*=.87), GAD-7 (*P*=.54), and DASS-21 (*P*=.71). We therefore assumed that the data were MCAR. The *t* tests and chi-squares were performed on those with missing data at 8 weeks and 3 months versus those with no missing data for demographics, program type, and baseline scores on each of the outcome measures. There were no statistically significant differences between groups on any baseline variables tested.

### Baseline Characteristics

The sample of 102 participants was mostly female (75/102, 73.5%), white (81/102, 79.4%), and in the 18 to 21 age group (56/102, 54.9%). A small percentage were international students (12/102, 11.8%). Most were undergraduate students (61/102, 59.8%), working part-time (51/102, 50.0%), and single or dating (66/102, 64.7%). Full baseline characteristics are reported in Table 1.

**Table 1:** Baseline characteristics of study sample.

Characteristic		Total sample (N=102), n (%)	*Space from depression* (n=31), n (%)	*Space from anxiety* (n=52), n (%)	*Space from stress* (n=19), n (%)
**Age group (years)**					
	18-21	56 (54.9)	17 (55)	28 (54)	11 (58)
	22-30	37 (36.3)	11 (35)	19 (36)	7 (37)
	Over 30	9 (8.8)	3 (10)	5 (10)	1 (5)
**Gender**					
	Female	75 (73.5)	20 (65)	39 (75)	16 (84)
	Male	27 (26.5)	11 (35)	13 (25)	3 (16)
**Student status**					
	Undergraduate	61(59.8)	18 (58)	31 (60)	12 (63)
	Graduate	41 (40.2)	13 (42)	21 (40)	7 (37)
**International student status**					
	United States	90 (88.2)	28 (90)	47 (90)	15 (79)
	International	12 (11.8)	3 (10)	5 (10)	4 (21)
**Employment**					
	Unemployed	34 (33.3)	10 (32)	16 (31)	8 (42)
	Part-time	51 (50.0)	16 (52)	26 (50)	9 (47)
	Full-time	17 (16.7)	5 (16)	10 (19)	2 (11)
**Relationship status**					
	Single or dating	66 (64.7)	22 (71)	34 (65)	10 (53)
	Committed relationship or married	36 (35.3)	9 (29)	18 (35)	9 (47)
**Ethnicity**					
	European American or white	81 (79.4)	25 (81)	40 (77)	16 (84)
	Asian or Asian American	8 (7.8)	4 (13)	3 (6)	1 (5)
	Hispanic or Latino	6 (5.9)	1 (3)	4 (8)	1 (5)
	African American or black	3 (2.9)	1 (3)	2 (4)	0 (0)
	Other	4 (3.9)	0 (0)	3 (6)	1 (5)

### Acceptability

The mean number of log-ins per participant was 14.3 (SD 12.1), with an interquartile range (IQR) of 4 to 21. Mean total time spent on the platform was 295.7 min (SD 377.3), IQR of 71.15 to 325.8, and the mean time spent using the platform per session was 27.4 min (SD 33.8), IQR of 8 to 30.8. The mean number of modules completed was 4.4 (SD 2.6), IQR of 2 to 7, and the mean number of journal entries was 6.0 (SD 8.2), IQR of 1 to 7. Participants completed a mean of 53.4% (SD 37.6), IQR of 18.1 to 98.6, of the total program content.

### Effectiveness

The linear model showed a significant decrease in mean PHQ-9, GAD-7, and DASS-21 stress subscale scores from baseline to 8 weeks, and from baseline to 3 months. The overall effect for depression was *F*_4_=6.36, *P*<.001, the overall effect for anxiety was *F*_4_=7.97, *P*<.001, and the overall effect for stress was *F*_4_=8.50, *P*<.001.

Table 2 shows the means and SDs for each of the 3 measures at all 3 time points, along with the effect sizes (Cohen *d*) comparing 8-week and 3-month scores with baseline scores for the completer sample.

### Analysis by Program Type

**Figure 1:**
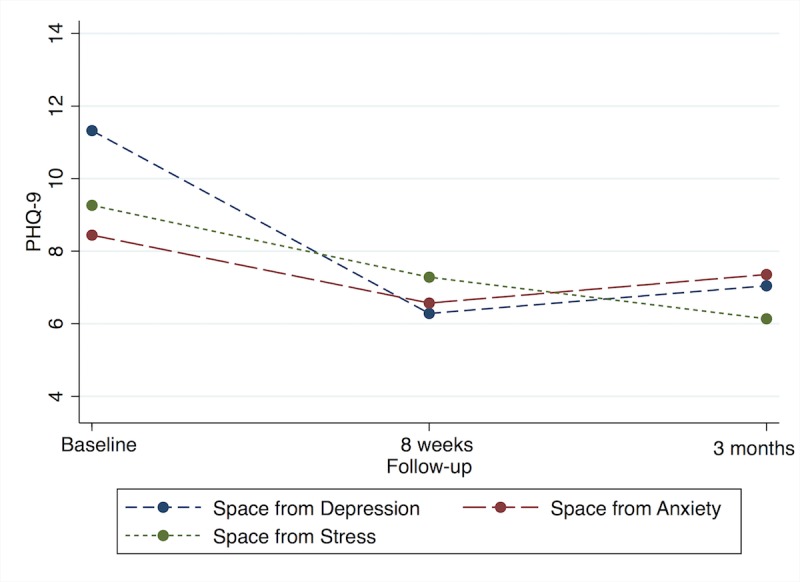
Linear mixed model adjusted Patient Health Questionnaire 9 (PHQ-9) scores over time, by program type.

The mixed-effects linear model for depression as measured by the PHQ-9 (Figure 1) showed highly significant changes in scores over time for those who chose the *Space from Depression* program (*F*_2,6_=13.7, *P*<.001), with decreases from baseline to 8-week (mean difference 5.0, 95% CI 3.0-7.1) and 3-month (mean difference 4.3, 95% CI 2.1-6.4) follow-up. Those who chose the *Space from Anxiety* program had nonsignificant decreases in PHQ-9 scores over time (*F*_2,6_=2.4, *P*=.09), with scores at 8 weeks (mean difference 1.9, 95% CI 0.2-3.6) and 3 months (mean difference 1.1, 95% CI −0.7 to 2.9) decreasing slightly. The decrease in depression symptoms in those who chose the *Space from Stress* program was significant (*F*_2,6_=3.0, *P*=.04), with decreases at 8 weeks (mean difference 2.0, 95% CI −0.7 to 4.7) and 3 months (mean difference 3.1, 95% CI 0.3-5.7).

**Figure 2:**
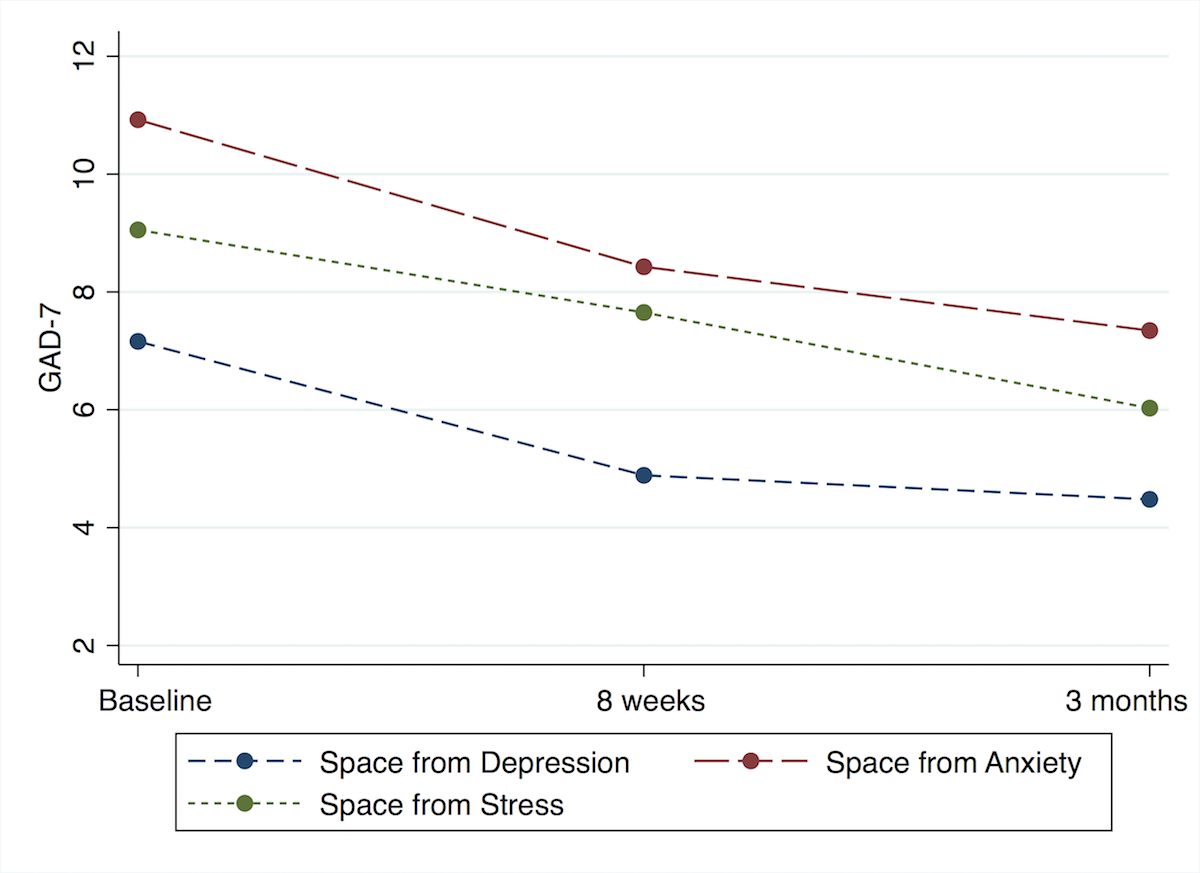
Linear mixed model adjusted Generalized Anxiety Disorder 7 (GAD-7) scores over time, by program type.

The GAD-7 scores for all 3 program types significantly decreased over time according to the LMM (Figure 2). The largest effect was among those who chose the *Space from Anxiety* program (*F*_2,6_=13.6, *P*<.001), with decreases in scores at 8 weeks (mean difference 2.5, 95% CI 1.1-3.9) and 3 months (mean difference 3.6, 95% CI 2.1-5.0). Those in the *Space from Depression* program had a significant decrease over time (*F*_2,6_=5.9, *P*=.01), with scores decreasing at 8 weeks (mean difference 2.6*,* 95% CI 0.3-3.9) and 3 months (2.7, 95% CI 1.0-4.4). Finally, those in the *Space from Stress* program also showed significant decreases over time (*F*_2,6_=4.3, *P*=.01) and lower scores at both 8 weeks (mean difference 1.4, 95% CI 0.7-3.6) and at 3 months (mean difference 3.0, 95% CI 1.0-5.0).

**Figure 3:**
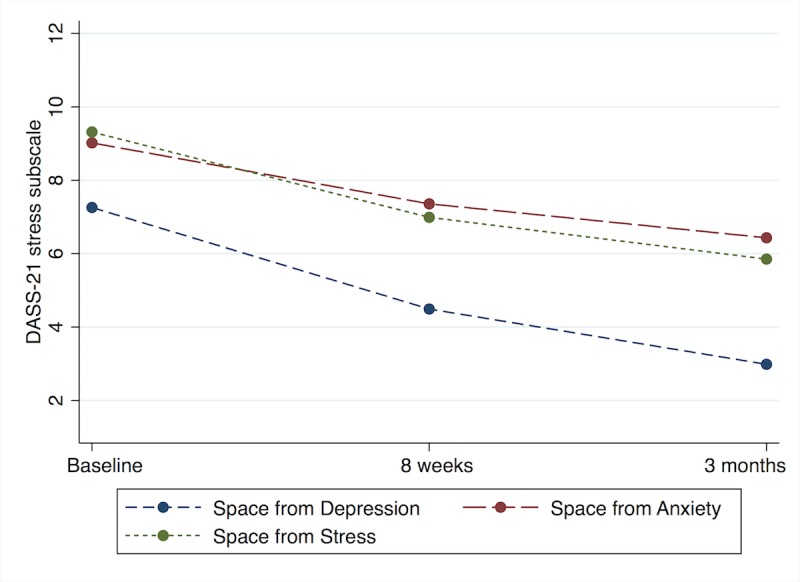
Linear mixed model adjusted Depression Anxiety and Stress Scale (DASS-21; stress subscale) scores over time, by program type.

The DASS-21 stress scores in the linear model also showed significant decreases over time among all program types (Figure 3). *Space from Stress* (*F*_2,6_=6.0, *P*=.01) users had decreased stress scores at both 8 weeks (mean difference 2.3, 95% CI 0.2-4.5) and 3 months (mean difference 3.5, 95% CI 1.4-5.5). *Space from Anxiety* (*F*_2,6_=6.7, *P*=.01) users decreased their scores at 8 weeks (mean difference 1.7, 95% CI 0.3-3.0) and 3 months (mean difference 2.6, 95% CI 1.1-4.0). Finally, *Space from Depression* (*F*_2,6_=12.8, *P*<.001) users also had decreased scores at both 8 weeks (mean difference 2.8, 95% CI 1.1-4.4) and 3 months (mean difference 4.3, 95% CI 2.6-6.0).

The means and SDs by program type, for baseline through 3 months, can be seen in Table 3, along with the effect sizes (Cohen *d*) for the difference between 8-week and 3-month means compared with baseline for the completer sample.

### Reliable Change

The RCIs for each of the 3 outcome measures were 5.38 for PHQ-9, 3.49 for GAD-7, and 4.88 for DASS-21.

As measured by the PHQ-9, among the 53 participants with 8-week follow-up data, 30% (16/53) decreased their scores by more than the RCI (6+), and thus had reliable change; 68% (36/53) did not have reliable change; and 2% (1/53) had reliable deterioration (increase of 6 of more). Of the 50 participants with 3-month data, 30% (15/50) had reliable change, 62% (31/50) had no reliable change, and 8% (4/50) had reliable deterioration.

**Table 2:** Mean (SD) scores for the Patient Health Questionnaire 9 (PHQ-9), the Generalized Anxiety Disorder 7 (GAD-7) questionnaire, and the stress subscale of the Depression, Anxiety, and Stress Scale (DASS-21) at baseline, 8 weeks, and 3 months.

Outcome measure	Baseline, mean (SD)	8 week, mean (SD)	Cohen *d* (95% CI)	3 month, mean (SD)	Cohen *d* (95% CI)
PHQ-9	9.5 (5.1)	6.5 (4.1)	0.59 (0.14-0.92)	7.1 (4.8)	0.46 (0.06-0.83)
GAD-7	9.4 (4.6)	6.9 (4.8)	0.58 (0.20-0.98)	6.1 (4.1)	0.82 (0.38-1.17)
DASS-21	8.5 (4.3)	6.3 (4.2)	0.49 (0.10-0.87)	5.2 (3.9)	0.77 (0.34-1.13)

**Table 3:** Mean (SD) scores for the Patient Health Questionnaire 9 (PHQ-9), the Generalized Anxiety Disorder 7 (GAD-7) questionnaire, and the stress subscale of the Depression, Anxiety, and Stress Scale (DASS-21) at baseline, 8 weeks, and 3 months, in users of *Space from Depression* program, *Space from Anxiety* program, and *Space from Stress* program.

Outcome measure		Baseline, mean (SD)	8 week, mean (SD)	Cohen *d* (95% CI)	3 month, mean (SD)	Cohen *d* (95% CI)
***Space from Depression* program**		**n=31**	**n=18**		**n=16**	
	PHQ-9	11.3 (5.8)	6.2 (4.3)	0.84 (0.06-1.41)	7.3 (6.5)	0.54 (0.08-1.25)
	GAD-7	7.2 (4.3)	5.4 (4.8)	0.44 (0.02-1.13)	5.2 (3.6)	0.56 (0.14-1.18)
	DASS-21	7.3 (4.8)	4.8 (3.2)	0.49 (0.24-1.07)	3.4 (2.2)	1.12 (0.15-1.60)
***Space from Anxiety* program**		**n=52**	**n=25**		**n=22**	
	PHQ-9	8.4 (4.4)	6.1 (4.1)	0.60 (0.02-1.15)	7.3 (4.3)	0.28 (0.27-0.84)
	GAD-7	10.9 (4.3)	7.5 (4.9)	0.74 (0.21-1.36)	6.7 (4.6)	0.94 (0.39-1.56)
	DASS-21	9.0 (3.9)	6.8 (4.6)	0.51 (0.00-1.12)	6.0 (4.9)	0.75 (0.24-1.47)
***Space from Stress* program**		**n=19**	**n=10**		**n=12**	
	PHQ-9	9.3 (5.0)	7.9 (4.0)	0.30 (−0.60 to 1.16)	6.3 (2.8)	0.74 (−0.27 to 1.49)
	GAD-7	9.0 (4.2)	8.0 (4.2)	0.32 (−0.48 to 1.12)	6.3 (4.1)	0.88 (0.03 to 1.71)
	DASS-21	9.3 (4.1)	7.4 (4.5)	0.49 (−0.37 to 1.40)	5.9 (3.0)	1.16 (0.17 to 1.87)

On the GAD-7, among those with 8-week follow-up data, 17 (17/53, 32%) decreased their scores by more than the RCI (4+), and thus had reliable change; 30 (30/53, 57%) had no reliable change; and 6 (6/53, 11%) had reliable deterioration (increase of 4 of more). At 3 months, 26 (26/50, 52%) had reliable change, 22 (22/50, 44%) had no reliable change, and 2 (2/50, 4%) had reliable deterioration.

Finally, as measured by the DASS-21 stress subscale, 31% (16/53) achieved reliable change (decrease of 5+) at 8 weeks and 42% (21/50) did so at 3 months. In contrast, 6% (3/53) and 4% (2/50) reliably deteriorated (increasing symptoms by 5 or more points) at 8 weeks and 3 months, respectively. In total, 34 (34/53, 64%) had no reliable change at 8 weeks, and 27 (27/50, 54%) had no reliable change at 8 weeks and 3 months, respectively.

### Satisfaction

The SAT questionnaire was included as part of the 8-week follow-up and, thus, given to all 53 participants who responded to the other follow-up questionnaires at 8 weeks. In total, 4 quantitative questions produced the following results: (Q1) “I was happy to use the computer to access treatment”: 83% (44/53) agreed or strongly agreed, 13% (7/53) were neutral, 4% (2/53) disagreed; (Q2) “I found the online treatment easy to use”: 82% (43/53) agreed or strongly agreed, 13% (7/53) were neutral, 5% (3/53) disagreed; (Q3) “I feel the treatment received will have a long lasting effect”: 45% (24/53) agreed or strongly agreed, 38% (20/53) were neutral, and 17% (9/53) disagreed or strongly disagreed; and (Q4) “Please rate how helpful you found the online treatment programme”: 69% (37/53) found it helpful or very helpful, 27% (14/53) found it not really or not at all helpful, and 4% (2/53) had no opinion.

On the 2 open-ended questions, participants reported different helpful aspects of the iCBT intervention, which were grouped into 3 categories: the delivery format (n=16), the content and CBT tools (n=13), and the supporter (n=1). In addition, participants indicated a number of unhelpful aspects grouped into the categories of format of support (n=8), lack of tailoring (n=6), and internal factors (n=4).

With regard to *delivery format*, participants reported the Web-based format to be helpful owing to convenience, flexibility, time management, access, and feeling in control of the pace of the use of the intervention. The following user quotes support these aspects of the format of delivery:

I could do it on my time when I wanted to. If I felt anxious or nervous or I had a bad day, I could just log on for a few minutes and focus on myself.

As a student it makes it easier to focus on my mental health in my free time. Scheduling mental health appointments can be stressful, but I can do this whenever and it just makes it a lot easier.

In addition, aspects of the *content of the intervention* and specific CBT tools were identified as helpful to participants. In general, participants noted how they learned from the intervention and that they used various tools to learn new skills. Specifically, participants indicated that the mood monitor, activity scheduling, and journal were particularly helpful tools:

I really liked the online journal, which is what I mostly used to express my feelings.

I feel like I learned a lot more about each CBT technique than I did when I was enrolled in in-person counseling

The *Supporter* was also important in the delivery of the intervention:

I like supporter’s review the most. The fact that some professional is willing to invest their time in following my activities for the treatment means a great deal to me.

Participants found 2 aspects of the *format in which the support was delivered* to be unhelpful. The first was that in-person counseling would have been preferred:

The fact that it’s online. I personally prefer in-person meetings.

I felt like it was more difficult to convey how I was feeling on an online platform than it would have been in in-person counseling. This was due to a lack of a connection I felt with my supporter [in comparison to in-person counseling] and because there is only so much I could convey in writing [in comparison to face-to-face conversations].

The second *unhelpful* theme focused on the reviews that supporters gave to participants:

I was hoping it would be a little more interactive, where I would have more feedback with a therapist about my individual problems and things that are specific to me.

Some participants reported a *lack of tailoring* to their specific needs. For example, the content was perceived to be too generic or not applicable for those who had done CBT before:

Some of the activities were hard or didn’t really apply to my situation.

Finally, *internal factors,* including lack of motivation and being busy contributed to participants finding the experience unhelpful:

Not quite as good at holding me accountable for doing it as an actual appointment.

Not a ton of motivation to finish a session every week, sometimes I would forget. Maybe reminder emails or an app would be helpful?

### Structured Interviews

The structured interviews provided further information regarding how students perceived the SilverCloud programs. Their responses are grouped into 3 categories: accessing SilverCloud, specific aspects of SilverCloud, and satisfaction with the SilverCloud programs.

A total of 14 structured interviews were conducted. Those who completed interviews were similar to the overall sample, in that they were mostly female: 71% (10/14) versus 73.5% (75/102) and mostly white: 86% (12/14) versus 79.4% (81/102), although a much larger percentage were graduate students: 71% (10/14) versus 40.2% (41/102). In total, 14% (2/14) were international students, a similar percentage to the overall sample (11.8%, 12/102). The mean baseline PHQ-9 score was 10.3 (SD 4.5), and the mean baseline GAD-7 score was 10.6 (SD 4.9), both of which were similar to the baseline scores of the overall sample (9.5 and 9.4, respectively). The mean baseline DASS-21 stress score was 9.6 (SD 4.2), similar to the overall sample (8.5). Similar to the whole sample, 64% (9/14) completed 8-week follow-up measures (compared with 52.0% (53/102) in full sample), and 50% (7/14) completed 3-month follow-up measures (49.0%, 50/102 in full sample). The mean PHQ-9 scores at 8 weeks and 3 months were 7.8 (5.0) and 7.6 (4.2), respectively (6.5 and 7.1 in the full sample), mean GAD-7 scores were 6.6 (3.7) and 6.7 (2.9), respectively (6.9 and 6.1 in the full sample), and mean DASS-21 stress scores were 7.0 (3.4) and 5.0 (2.6), respectively (6.3 and 5.2 in the full sample).

### Accessing SilverCloud

The interviewer asked students how they found out about the program and why they chose to use it. Most students found out about the intervention on the internet (11/14, 79%), in the process of looking for mental health treatment. All the participants mentioned scheduling and convenience as a reason for using the Web-based programs over in-person therapy. One student, for example, mentioned choosing SilverCloud:

...because my schedule is very busy and I wasn’t really sure whether I wanted to do counseling or not, so it felt like a good way to kind of check it out without much commitment.

Some also noted that they did not feel comfortable with the idea of in-person counseling, citing worries about confidentiality and intensity (4/14, 29%). Only 1 had done anything like it before.

Students were also asked when they used the program. Their responses were divided: some scheduled specific times for it (10/14, 71%); some used it when they were feeling depressed, anxious, or stressed (8/14, 57%); and others worked on it when they had free time (4/14, 29%). For those who used it on more than one of these occasions (n=7), there were mixed results in terms of when it was perceived to be most effective. Some described being able to get more out of it when they were not distressed, as they could approach it with a clear mind:

I would say when I actually sat down to do it just to complete it, that actually was more helpful than doing it when I was down, because when I was down I was looking for answers, whereas I was in a more rational and clear mindset when I was just doing it out of my own.

Others noted that it was most helpful when they were feeling distressed:

It probably felt more helpful when I was coming off of a particularly stressed time, or like when I felt like a serious failure or that sort of thing. So yeah, when I felt worse, it probably helped me a little bit more than doing it on a day when I felt totally fine.

One student noted that it was particularly helpful to journal when they were feeling distressed but that they read the informative parts of the modules when they were not feeling distressed.

### Specific Aspects of SilverCloud

In general, students appreciated having choices about different aspects of the intervention. For example, students were asked what they thought about the ability to complete the intervention in any order. Most enjoyed this about the program (13/14, 94%) because it allowed them to access the parts they wanted and also allowed them to go back and engage with previous parts of the program that they found helpful. However, 1 was unaware that it could be completed in any order, and others completed it in order anyway (3/14, 21%). Students generally reported feeling positive about having a choice about whether they used the stress, anxiety, or depression program (8/14, 57%), although some would have preferred to be assigned one (2/14, 14%), and some were confused about the process (2/14, 14%), with 1 student noting:

I just kind of chose one at random, I wasn’t really sure which one to choose, and then, I don’t know if they have practically different programs, or if there, if they have some overlap, I’m not sure.

One student specifically expressed that they would have liked to access multiple programs.

The interview also included questions about having a supporter. Almost all participants appreciated having a supporter (13/14, 94%), and some noted that they specifically liked receiving feedback (5/14, 36%):

It kind of presented the opportunity to have someone to reach out to and ask some other questions. She gave me some really good guidance on what to do next step.

Some participants also said that the supporter helped them feel accountable (2/14, 14%):

Sometimes I feel like I wouldn’t have gone on or I was really lazy and didn’t really do anything, so I felt really guilty when she would take the time, like my supporter would take the time to look over everything and that I didn’t like do anything. So I felt bad sometimes. But I liked having that.

A large portion (11/14, 79%) of the participants would have liked more from the supporter. Participants specifically wanted more detailed and personalized feedback and felt like they lacked a connection with the supporter. Some participants would have liked to be able to speak directly with their supporter over the phone or video chat service:

Personally, I think that I was hoping for a little bit more of a personalized approach, and that maybe I would get more feedback about me personally and my issues from the person that was assigned to me. It was a lot more general feedback, like I could tell she [the supporter] had read the things I had done, but she wasn’t there, you know, to talk to me about my problems. So I guess I was expecting it to be a little bit more communication based.

### Satisfaction With SilverCloud Programs

When asked how they felt about using SilverCloud, some participants noted that they felt positive about the program and that it met their needs (6/14, 43%), some expressed more mixed feelings (6/14, 43%), and others did not find it helpful (2/14, 14%). Some students with mixed feelings reported that it was helpful to some degree but thought that they would need to seek other treatment to more fully address their concerns. Another student noted that they learned a lot but had not yet noticed a change in symptoms.

Those who had experience with in-person therapy (8/14, 57%) were asked how that experience compared with using SilverCloud. They noted strengths and weaknesses of both. In-person therapy was described as more personalized, specific, and flexible in terms of content. Some specifically noted that SilverCloud could not replace in-person therapy:

I think there’s something in-person offers you when it becomes so much more personalized, that SilverCloud just can’t reach.

One student noted that they felt like the program would have been more helpful for them if they had not already done in-person therapy. Participants generally noted that SilverCloud was more flexible in terms of time and was less intense than in-person therapy, and 1 person specifically noted that they liked SilverCloud better than in-person therapy, explaining that:

...while it was nice having the one-on-one time, I think specifically for me, having the information all set there that I could read and kind of understand on my own terms was very very nice.

When directly asked, most participants (11/14, 79%) had positive reactions to the idea of completing SilverCloud in conjunction with in-person therapy, as this would help address the issue of personalization. Some suggested that they would like to see their supporter in person infrequently while using SilverCloud in between sessions:

I think that that would be more beneficial than how it is right now. Like with the therapist, if you had an initial in-person session, and then you had like a few, like several online sessions before you went back again, I feel like um that would alleviate a lot of the problems you’re facing. And maybe they could personalize the material that they like sent you between sessions as well.

One student noted that, having used SilverCloud, they would be more likely to try in-person therapy. One participant sought additional mental health treatment while they used SilverCloud.

## Discussion

### Key Findings

The aims of this mixed-methods study were to assess feasibility, acceptability, effectiveness, and satisfaction with iCBT interventions embedded within the care delivery system of a large university. Below, we discuss key findings along with limitations and future directions.

The feasibility of offering the iCBT interventions as part of service delivery at the university was evaluated primarily in terms of the number of students who expressed interest and began one of the programs. Approximately 100 students began a program, which was 56% of those who received an invitation and brief overview. Thus, it is feasible to incorporate iCBT into service delivery. It is important to note that most students who were referred to iCBT were seeking in-person counseling; the number recruited might be higher among students who were not already seeking this kind of care. In addition, more students likely would have been recruited if we had launched a larger marketing campaign (eg, posters in dorms and around campus and mass emails). It is not clear whether fewer students were recruited from the international student office because of cultural differences related to the acceptability of help seeking or some other reason, although that is something to explore in future research.

Most students chose the *Space from Anxiety* program, even among those who scored above our threshold for moderate symptoms on all 3 measures. They were much less likely to choose the *Space from Stress* program. This might reflect students’ perception of their own symptoms and which seem most urgent or best addressed through a Web-based program. Other iCBT courses offer patients a choice of programs such as the MindSpot clinic (offering well-being, well-being plus, obsessive compulsive disorder, and posttraumatic stress disorder programs) [29]; however, their well-being programs combine depression and anxiety. Other studies have observed a relationship between patient choice and improved outcome [30], but the reasons *why* a particular program was chosen over another and the balance between choice and clinical recommendation should be explored further to optimize treatment outcomes. This is especially important given the overlap of depression, anxiety, and stress symptoms.

Acceptability was assessed in terms of the extent to which participants used the program after they began. In total, participants navigated through approximately 50% of the program content during an average of 14 sessions lasting approximately 5 hours in total. One systematic review found similar results, with users completing 50% to 70% of the content in Web-based interventions [31]. A possible reason behind the completion rates might be that users who have obtained a desired outcome stop usage altogether. However, the programs tested here are constructed in a nonlinear way, and not all of the specific content needs to be accessed for the modules or program to be completed or for the benefit achieved from each module to be obtained. Nevertheless, it would be worthwhile to identify usage thresholds needed to achieve the biggest effect on desired outcomes in future research. This is important as usage can vary substantially from person to person, as exemplified in our sample. For example, although 18 students did not post a single journal entry, 9 posted more than 20. Pinpointing the ideal cut-off for the usage variables is key to maximizing the potential of Web-based interventions.

With regard to effectiveness, symptoms of depression, anxiety, and stress decreased over time in users of all 3 programs, with small (0.3) to very large (1.2) within-group effect sizes. The results are in line with previous research on the SilverCloud *Space from Depression* program [32]. A recent randomized controlled trial (RCT) [33] measuring the effects on stress of a Web-based and app-based intervention among college students found similar medium effect sizes (0.59 at 7 weeks) to the ones reported in our study for the stress subscale of the DASS-21 at 8 weeks (0.49-0.51), although our study had larger (0.75-1.16) effect sizes at 3-month follow-up (vs a 3-month effect size of 0.67 in this RCT). However, there was an increase in PHQ-9 symptoms from 8 weeks to 3 months in users of the *Space from Depression* and *Space from Anxiety* programs, whereas *Space from Stress* users continued to show decreases in PHQ-9 scores from 8 weeks to 3 months. This suggests the latter was best at maintaining low depressive symptoms, although larger trials are needed to replicate these findings.

In addition, a recent meta-analysis of 48 studies assessing the effectiveness of universal and indicated prevention approaches to delivering technology-based mental health intervention to college students found that the overall mean between-group effect sizes for indicated interventions (0.37) was larger than the overall mean between-group effect size for universal approaches (0.19) [10]. Although both effects were significant, these results favor an indicated prevention approach. This meta-analysis also indicated that Web-based interventions with some form of additional support such as prompts, feedback, or guidance through emails, further improved the outcomes for indicated interventions (*d*=0.55) [10]. Overall, these results are similar to the findings of this study and support an indicated approach to Web-based mental health interventions and suggest the benefit of providing clinician support within the framework of the intervention.

The reliable change analyses indicated that approximately one-third of the users had reliable change on the outcome measures at 8 weeks. Slightly higher percentages of participants achieved reliable change at 3 months (30%-52% across measures). These results positively reflect on the clinical utility of the interventions and their ability to maintain clinical changes beyond the acute treatment period. However, slightly fewer subjects completed the measures at the 3-month follow-up, and it is unclear if those who dropped out maintained their gains. As the RCI analyses excluded participants with missing data, there is a risk of bias in these results. However, the reliable change results are similar to other research on SilverCloud [32], also using per-protocol analysis and previous work in the field of internet-delivered interventions. For instance, clinical recovery rates between 25% and 49% have been reported [33,34,12].

Finally, participants’ responses to the satisfaction questionnaire and the interviews suggested several strengths and limitations of the programs. Students generally found the programs to be helpful, with few saying that they found them unhelpful. Factors mentioned as helpful included flexibility, convenience, and having control over the pace of the intervention. Some students noted that they found the content of the interventions helpful, along with the tools available to learn new skills. In contrast, other students found that the content was not tailored enough to their specific needs. Participants also felt that having a supporter was helpful, although many wanted more contact with and feedback from the supporter. One solution is to offer students the opportunity to complete iCBT interventions along with less frequent (eg, monthly) in-person therapy; more research is needed to determine the feasibility and effectiveness of such a delivery format. Finally, despite having a supporter, some found it difficult to stay motivated to complete the program on their own, especially given busy schedules. Taken as a whole, the qualitative results provide a more nuanced and comprehensive understanding of the program, which can be used to guide future research on how to improve Web-based interventions.

These results are consistent with another qualitative study of students’ perceptions of Web-based interventions [14]. In both studies, participants used Web-based interventions because of the scheduling flexibility. In addition, participants from both studies called for interventions that were more personalized to their specific needs and life circumstances. Participants in the Fleischmann et al’s study wanted greater flexibility in terms of the order in which they completed the program, and participants in this study spoke positively of that flexibility. Future studies should examine whether this flexibility actually increases adherence, effectiveness, and satisfaction.

### Limitations and Future Directions

This study also has limitations, including the lack of a control group and the lack of follow-up data on a sizable portion of the initial sample. Without a control group, we cannot be sure that decreases in symptoms were because of the intervention rather than the natural course of symptoms over time. As the purpose of this study was to integrate Web-based interventions into natural service delivery, to randomize help-seeking students into a placebo arm would have been difficult. Our conclusions regarding the effectiveness of the interventions would be strengthened by a larger RCT. The lack of follow-up data could be because of several factors. Supporters might not have encouraged the continued use of the program by study participants strongly enough, in some cases. Some study participants might have dropped out because of unmet expectations with regard to their Web-based program, and some might have completed the program early and met all their needs before the 8-week period. The rates of missing data at follow-up are similar to previous studies, and indeed younger persons are more likely to drop out than older participants in studies on self-managed internet interventions [35]. Although it is not possible to determine if the missing data positively or negatively affected the results, because the data appeared to be missing completely at random and individuals with missing data were no different as a group than those without missing data, the likelihood of bias is reduced. Future trials could attempt to follow-up with those dropping out altogether, as the reasons for dropout are important to understand who benefits most from these interventions and what personal characteristics predict positive outcomes. Larger, controlled studies are warranted to replicate these findings and to assess whether improving adherence improves outcomes.

### Conclusion

In conclusion, we echo the call that Web-based interventions are a useful addition to the list of solutions for addressing growing mental health service needs on campus [6]. Overall, the iCBT programs tested in our study appear to be feasible, acceptable, and effective in a university environment. Participants described the benefits of having a flexible, supported Web-based intervention available on campus. Larger trials should be conducted to further test the significance of supported Web-based interventions that also give students a choice of program depending on their symptom profiles. Improving the delivery and reach of these programs has the potential to positively affect students’ mental health at this key life stage.

## Appendix

Multimedia Appendix 1 Study CONSORT diagram.

## Abbreviations

ANOVA: analysis of variance
CBT: cognitive behavioral therapy
GAD-7: Generalized Anxiety Disorder 7
DASS-21: Depression Anxiety and Stress Scale
DSM-5: Diagnostic Manual of Mental Disorders, Fifth Edition
iCBT: internet-delivered cognitive behavioral therapy
IQR: interquartile range
LMM: linear mixed model
MCAR: missing completely at random
PHQ-9: Patient Health Questionnaire 9
RCI: reliable change index
RCT: randomized controlled trial
SAT: Satisfaction With Treatment
