# Supplementary figures and images for: Supported Internet-Delivered Cognitive Behavioral Therapy Programs for Depression, Anxiety, and Stress in University Students: Open, Non-Randomised Trial of Acceptability, Effectiveness, and Satisfaction
Source: JMIR Ment Health. 2018 Dec 14;5(4):e11467. doi: 10.2196/11467 (PMC6315236; doi:10.2196/11467)

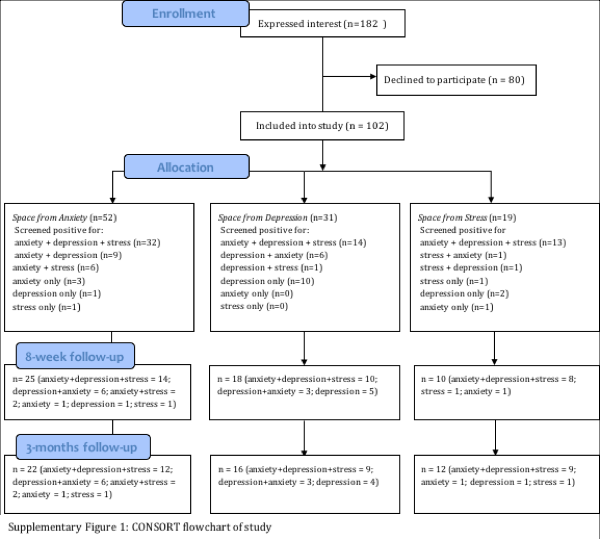

Supplement: Multimedia Appendix 1 [file mental_v5i4e11467_app1.png]
